# Circulating Th17.1 cells as candidate for the prediction of therapeutic response to abatacept in patients with rheumatoid arthritis: An exploratory research

**DOI:** 10.1371/journal.pone.0215192

**Published:** 2019-11-20

**Authors:** Shinji Maeda, Satoshi Osaga, Tomoyo Maeda, Norihisa Takeda, Shin-ya Tamechika, Taio Naniwa, Akio Niimi

**Affiliations:** 1 Department of Respiratory Medicine, Allergy and Clinical Immunology, Nagoya City University Graduate School of Medical Sciences, Nagoya, Japan; 2 Clinical Research Management Center, Nagoya City University Hospital, Nagoya, Japan; Nippon Medical School, JAPAN

## Abstract

T-helper (Th)17.1 cells exhibit high pathogenicity in inflammatory diseases. This study aimed to identify the changes in the proportions of Th subsets, including Th17.1, which are associated with abatacept treatment response in Japanese patients with rheumatoid arthritis. On the basis of the results, we assessed whether Th17.1 is a potential cellular biomarker. Multicolor flow cytometry was used to determine the circulating Th subsets among CD4+ T lymphocytes in 40 patients with rheumatoid arthritis before abatacept treatment. All the patients received abatacept treatment for 24 weeks; changes in disease activity score, including 28-joint count C-reactive protein, and responsiveness indicated by other indices to abatacept treatment were evaluated according the European League Against Rheumatism criteria (good and moderate responders and nonresponders). The correlation between the abatacept responses and the proportions of Th subsets (baseline) was analyzed. Logistic regression analysis with inverse probability weighting method was performed to calculate the odds ratio adjusted for patient characteristics. The proportion of baseline Th17.1 cells was significantly lower in patients categorized as good responders than in those categorized as non-good responders (moderate responders and nonresponders; p = 0.0064). The decrease in 28-joint count C-reactive protein after 24 weeks of abatacept therapy showed a significant negative correlation with the proportion of Th17.1 cells. The adjusted odds ratio for achieving good response in patients with baseline Th17.1 levels below the median value was 14.6 (95% confidence interval, 2.9–72.3; p = 0.0021) relative to that in the remaining patients. The proportion of Th17.1 cells at baseline is a good candidate for predicting abatacept treatment response in Japanese patients. These novel findings may represent a significant step in the pursuit of precision medicine.

## Introduction

Advances in medicine and pharmaceutical technology have led to tremendous improvements in the treatment of rheumatoid arthritis (RA) [1]. Moreover, new autoimmune cells have been found through research in clinical human immunology. The development of potent antirheumatic drugs, particularly biological products, has helped improve clinical remission rates [2,3]. The targets of these biologics include cytokines and T cells, both of which play key roles in RA pathogenesis. The therapeutic effect of abatacept (ABA), a strong inhibitor of T cells [4], has been shown to be equivalent to that of tumor necrosis factor-α (TNF-α) inhibitor therapy [5–7].

The target lymphocytes of ABA, particularly CD4+ T cells, play a central role in RA pathogenesis, particularly in terms of acquired immunity, and autoimmune response induction [4]. Studies conducted using autoimmune mouse models [8,9] have demonstrated the decisive role of T-helper (Th)17 cells in the pathogenesis of arthritis and autoimmune diseases. In humans, CCR6, which is a representative surface marker of Th17 [10], is a disease susceptibility gene of human RA [11]. Th17 is also involved in human RA pathology [12] through antigenic production of inflammatory cytokines, such as interleukin (IL)-17A, IL-17F, and IL-22. Unlike those in mice, these human Th17 cells have subpopulations. In particular, Th17.1 cells that are CD161+ CXC chemokine receptors (CXCR3)+ interferon-γ-producing Th17 cells, also referred to as nonclassical Th1 or extinguish Th17 (ex-Th17), are a subgroup of Th17 cells found in humans [13] and are believed to be the most pathogenic among the CCR6+ CD4+ T cells [12]. Th17 cells convert to inflammatory Th17.1 cells in an inflammatory milieu induced by cytokines, such as IL-1β, IL-23, TNF-α, and IL-12 [14]. Th17.1 cells have high expression levels of P-glycoprotein/multidrug resistance type 1 (MDR-1) and exhibit glucocorticoid resistance [13]. Thus, RA is characterized by joint destruction that is resistant to steroid treatment alone [15]. It is believed that the glucocorticoid resistance of Th17.1 cells is not attributable only to the function of MDR-1, and thus, further elucidation of the mechanism is expected in the future.

Although the therapeutic efficacy of ABA has been well demonstrated, some patients are refractory to ABA treatment. The immune mechanisms driving synovitis chronicity are multifactorial, such as adoptive and innate immune, stromal, and systemic pathways [16]. Therefore, the contribution of immune factors other than T cells may influence the efficacy of ABA therapy. Among the various T cell subsets, some decrease in numbers in response to ABA treatment, whereas others do not [17]. ABA treatment markedly reduces the proportion of T follicular helper cells and slightly decreases Th17 cells and activated regulatory T cells (Tregs). However, no studies have focused on the relationship between Th17.1 cells and therapeutic response to ABA in patients with RA. Moreover, the identification of drug-specific biomarkers that predict the therapeutic response is crucial and a desirable goal in the realm of personalized medicine [1]. Indeed, advances in cell analysis technology have raised prospects for the discovery of novel cellular immunological biomarkers that can predict treatment response in rheumatic diseases [18,19].

In the present study, we analyzed the proportion of each Th cell subset, including CXCR3+ Th17 cells (Th17.1), in the peripheral blood of Japanese patients with active RA before ABA treatment to explore the early cellular biomarkers of ABA treatment response. The correlation between the proportion of Th17.1 cells and ABA treatment response was demonstrated, and its potential use as a biomarker for predicting therapeutic response was proposed.

## Materials and Methods

### Ethics statement

This research was approved by the Ethics Review Committee of the Graduate School of Medicine, Nagoya City University. The study was conducted in accordance with the Declaration of Helsinki. Written informed consent was obtained from all patients.

### Participants

In Japan, patients with RA who met the criteria of the 1987 American College of Rheumatology RA classification (revised criteria of the RA classification) [20] and received ABA treatment at Nagoya City University Hospital between 2009 and 2015 were eligible for inclusion. The inclusion criteria were as follows: (1) patients who consented to participate in this research, (2) patients who agreed to provide peripheral blood mononuclear cells (PBMCs) for the immunophenotyping analysis of Th subsets and Treg using multicolor flow cytometry, and (3) patients who did not achieve adequate improvement on previous treatment with at least one conventional synthetic disease-modifying antirheumatic drug (DMARD).

Participants received intravenous ABA at 0, 2, and 4 weeks and every 4 weeks thereafter. The ABA dose was based on body weight (BW) as follows: 500 mg for patients with a BW of <60 kg and 750 mg for those with a BW of 60–100 kg.

### Cell surface and intracellular staining and flow cytometric analysis

After obtaining informed consent, we obtained the PBMCs of participants at baseline and at 4 and 24 weeks of ABA treatment. PBMCs were separated by density gradient centrifugation with Ficoll-Paque Plus (GE Healthcare, Uppsala, Sweden) and resuspended in flow cytometry buffer (Hanks’ Balanced Salt Solution supplemented with 2% heat-inactivated fetal calf serum, 0.05% sodium azide, and 0.5% EDTA). Cells were stained for 30 min at 4 °C under darkened conditions with the following fluorochrome-labeled monoclonal antibodies: anti-CD4-AmCyan (Clone SK3, BD Biosciences, Franklin Lakes, NJ, USA), anti-CD25-APCCy7 (Clone M-A251, BD Biosciences), anti-CD45RA-FITC (Clone HI100, BD Biosciences), anti-Ki67-FITC (Clone B56, BD Biosciences), anti-CD196 (CCR6)-PE-Cyanine7 (Clone R6H1, eBioscience, San Diego, CA, USA), anti-CD161-PE (Clone HP-3G10, eBioscience), anti-CCR4-Alexa647 (Clone TG6/CCR4, eBioscience), anti-CD183 (CXCR3)-Pacific Blue (Clone G025H7, BioLegend), anti-forkhead box P3 (Foxp3)-PerCP-Cyamine5.5 (Clone PCH101, eBioscience), rat immune(Ig)G2a-PerCP-Cyamine5.5 antibody (eBioscience), and anti-CD45RO-APC (Clone UCHL1, Tonbo Biosciences, San Diego, CA, USA). For the intracellular staining of Foxp3 and Ki67, the Foxp3 Staining Buffer Set (fixation/permeabilization and permeabilization buffers, eBioscience) was used according to the manufacturer’s protocol. Stained cells were washed twice using the flow cytometry buffer, resuspended for analysis using the FACSCanto II flow cytometer (BD Bioscience) and FACSDiva software (BD Bioscience), and analyzed with the FlowJo software (Tree Star). We defined the Th subset as follows:

Treg, CD4+ CD25+ Foxp3+; nonTreg, CD4+ Foxp3−; Th1, CXCR3+ CCR4− CCR6- nonTreg; Th2, CCR4+ CXCR3− CCR6− nonTreg; Th1&2, CXCR3+ CCR4+ CCR6− nonTreg; Th17, CCR6+ CD161+ CCR4+ CXCR3− nonTreg; and Th17.1, CCR6+ CD161+ CXCR3+ CCR4− nonTreg.

### Clinical assessment and evaluation of therapeutic response

Data pertaining to the following demographic and clinical variables were obtained from the medical records: age; sex; disease duration; use of corticosteroids, DMARDs, and nonsteroidal anti-inflammatory drugs; tender and swollen joint counts; patient and physician global assessments (patient and doctor’s visual analog scales, 0–100 mm); and C-reactive protein (CRP), matrix metalloproteinase-3 (MMP-3), rheumatoid factor (RF), and anti-citrullinated protein/peptide antibody (ACPA) levels.

Disease activity was assessed by calculating the disease activity score 28-joint count C-reactive protein (DAS28-CRP) for each patient at each visit. The DAS28-CRP was calculated, and patients were categorized into the following four groups: remission and low, moderate, or high disease activity (LDA, MDA, or HDA, respectively) according to the recommended formula (https://www.das-score.nl/das28/en/difference-between-the-das-and-das28/how-to-measure-the-das28/how-to-calculate-the-das28/alternative-validated-formulae.html). Because the DAS28-CRP values are reportedly lower than those obtained in the original DAS28 assessment using erythrocyte sedimentation rate, the cutoff values for HDA, LDA, and remission were the thresholds of 4.1 (instead of the original 5.1), 2.7 (instead of 3.2), and 2.3 (instead of 2.6), respectively [21]. The therapeutic response to ABA at 24 weeks was evaluated using the European League Against Rheumatism (EULAR) response criteria (https://www.das-score.nl/das28/en/difference-between-the-das-and-das28/importance-of-das28-and-tight-control/eular-response-criteria.html), with 4.1 and 2.7 as the thresholds for HDA and LDA, respectively. Briefly, patients were classified into three groups based on their 6-month DAS28-CRP and absolute change from baseline according to the EULAR criteria as no, moderate, or good response. A good responder (GR) must demonstrate an improvement of at least 1.2 units and achieve an absolute DAS28-CRP score of <2.7. A nonresponder must demonstrate an improvement of ≤0.6 and have a final DAS28-CRP score of >4.1. Moderate responses fall between these data points. Furthermore, responsiveness to ABA treatment was evaluated using the following indicators: the changes in disease activity before and after ABA treatment (ΔDAS28-CRP 0–24 weeks) and evaluation of disease activity after 24 weeks of ABA treatment (remission, LDA, MDA, HDA).

After the initiation of ABA therapy, the clinical course was followed up for 24 weeks (every 4 weeks), and the correlation between the responses to ABA treatment, RA disease activity, and baseline proportion of Th subsets among CD4+ T lymphocytes (before treatment) was analyzed.

### MDR-1 activity assay

For the analysis of the MDR-1 activity of T cells, the fluorescent dye rhodamine 123 (Rh-123) was used according to the methods reported elsewhere [13]. Briefly, total CD4+ T cells were isolated using the Dynabeads CD4 positive T cell isolation kit (Invitrogen). Purified cells were 95%–98% pure as determined by flow cytometric analysis. Purified T cells in complete medium (DMEM [Gibco] supplemented with 10% FBS, 1% L-glutamine, 1% sodium pyruvate, 1% HEPES, and 1% Pen-Strep [all from Gibco]) were loaded with Rh-123 (Sigma-Aldrich) at a final concentration of 1 μg/mL for 30 min on ice. Cells were then washed and moved to a 37 °C incubator for 2 h. After an efflux period, cells were washed on ice in PBS, stained with surface markers (CD4, CD45RO, CXCR3, CCR6, and CD161), and washed again in PBS, and stained cells were kept on ice prior to flow cytometric analysis. Fluorescence reduction due to the emission of fluorescent dye by MDR-1 was confirmed by flow cytometry. For a negative control, 1-μM cyclosporine A (Sigma-Aldrich) was added to cells immediately before the incubation step.

### Statistical analysis

The Mann–Whitney U and Fisher’s exact tests were used to assess between-group differences as regards continuous and categorical variables, respectively. The differences in continuous variables between three groups were analyzed using the Kruskal–Wallis test. Moreover, the Friedman rank sum and Wilcoxon signed rank tests were used to analyze sequential changes in the proportion of each Th subset among CD4+ lymphocytes (0, 4, and 24 weeks) and Ki67 expression in each Th subset (0 and 4 weeks). The correlation between two continuous variables was assessed using Spearman’s rank correlation coefficient. A stepwise variable selection method based on Akaike’s Information Criterion, Bayesian information criterion, and P value was performed to identify the candidate Th subset that predicted ABA response.

The enrolled patients (n = 40) were divided into two groups based on the median proportion of Th17.1 cells among CD4+ T cells: Th17.1 lower (n = 20) and Th17.1 higher (n = 20).

We performed leave-one-out cross validation to evaluate this Th17.1-ABA model’s generalizability to independent cohorts. We left one test case out of all cases, and the remaining 39 were training cases used to determine a cutoff value of Th17.1 to predict GR/non-GR (moderate responder or nonresponder) with ABA treatment by maximizing Youden’s index. The test case was used to validate whether the cutoff value correctly discriminates the ABA treatment response of the case. This process was repeated until all cases were left as test case, and a concordance rate between the actual and predicted responses and its kappa coefficient were calculated.

To minimize the potential confounding effect due to baseline differences in patient characteristics between the Th17.1-lower and Th17.1-higher groups, the inverse probability weighting (IPW) method, which is an application of the propensity score (PS) [22–24], was applied to compare the DAS28-CRP and good response rate of ABA treatment between the groups. The PSs for the IPW method were estimated using multivariate logistic regression analysis with the Th17.1 status (lower or higher) as the dependent variable and the following baseline characteristics as the independent variables: age, sex, DAS28-CRP (baseline), RF, ACPA RA disease duration (years), history of biological DMARDs, prescription of methotrexate (MTX), and glucocorticoids. The discriminative power of the PS was quantified by the C-statistic corresponding to the area under the receiver operating characteristic (ROC) curve. Next, each patient background variable was compared under the correction by the IPW method using the weighted Mann–Whitney and chi-square tests and weighted *t*-test, and the respective P values were calculated. The effect of Th17.1 on patient ABA response was evaluated using estimated odds ratios (OR) and 95% confidence intervals (CIs). All calculated P values were two-sided, and P values < 0.05 were considered statistically significant for all analyses. Statistical analyses were performed with the R software version 3.3.3 (R Development Core Team, Vienna, Austria) and EZR version 1.35 (Saitama Medical Center, Jichi Medical University, Saitama, Japan) [25], which is a graphical user interface for R (The R Foundation for Statistical Computing, Vienna, Austria). The following R software packages were used for statistical processing and creation of graphs and tables: survey (version 3.31–5) [26], aod (version 1.3), weights (version 0.85), ggplot2 [27], and corrplot (version 0.77).

## Results

### Baseline characteristics of patients

Table 1 shows the baseline demographics and clinical characteristics of the enrolled patients (N = 40). The disease activity of RA was high in the study population (median DAS28-CRP, 4.43; Simplified Disease Activity Index, 23.8). ACPA-positive patients accounted for 60% of the study population, and they were relatively older (median age, 70.5 years). With respect to the use of concomitant drugs, 77.5% of the patients were taking MTX, whereas 60% were taking glucocorticoids. With respect to medication history, only 32.5% of the patients had a history of treatment with biological DMARDs.

**Table 1:** Clinical characteristics of patients at baseline.

Patient characteristics	Overall n = 40 Median [IQR], or, (%)
Age, year old	70.5 [60.8, 74.6]
Sex: male/female, (%)	8/32 (20.0/80.0)
Disease duration (years)	4.2 [1.5, 15.9]
DAS28-CRP	4.43 [4.02, 5.01]
SDAI score	23.8 [20.3, 28.8]
CRP, mg/dL	1.02 [0.48, 2.27]
MMP-3 (ng/mL)	167.7 [100.2, 290.2]
ACPA (negative/positive, [%])	16/24 (40.0/60.0)
Negative, (%)	16 (40.0)
Low positive, (%)	5 (12.5)
High positive, (%)	19 (47.5)
RF (negative/positive, [%])	14/26 (35.0/65.0)
Negative, (%)	14 (35.0)
Low positive, (%)	9 (22.5)
High positive, (%)	17 (42.5)
Concomitant methotrexate, n (%)	31 (77.5)
MTX, mg (mean [sd])	9.4 (2.9)
Concomitant glucocorticoid, n (%)	24 (60.0)
Prednisolone, mg (mean, [sd])	5.8 (3.6)
Concomitant tacrolimus, n (%)	3 (7.5)
Concomitant NSAIDs, n (%)	20 (50.0)
Biologic DMARDs naïve, n (%)	27 (67.5)
Pulmonary complications associated with RA, n (%)	18 (45.0)

This table shows the patient baseline demographics. Data are presented as median (IQR, interquartile range), mean (SD), or frequency (%).

DAS28-CRP, disease activity score 28-joint count C-reactive protein; SDAI, Simplified Disease Activity Index; CRP, C-reactive protein; NSAIDs, nonsteroidal anti-inflammatory drugs; MMP-3, matrix metalloproteinase 3; ACPA, anti-citrullinated protein antibody; RF, rheumatoid factor; MTX, methotrexate; DMARDs, disease-modifying antirheumatic drugs; low positive, less than three times the normal upper limit among positive; high positive, more than three times the normal upper limit.

Between-group differences with respect to the median and percentage values were determined using the Mann–Whitney U and Fisher’s exact tests, respectively.

### Characterization of Th17.1 in patients with RA

**Fig 1:**
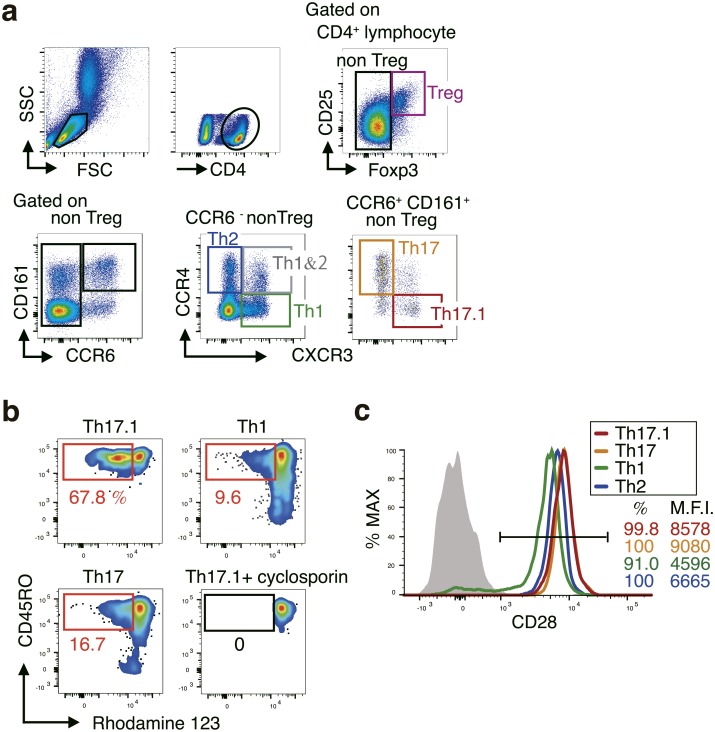
Characteristic of circulating Th17.1 cells in patients with rheumatoid arthritis (RA). a. Flow cytometry plots explaining the gating strategy for the identification of peripheral blood Treg, Th1, Th2, Th1&2, Th17, and Th17.1 subpopulations. CD4+ T cell subsets in the peripheral blood of adult patients with RA were analyzed using flow cytometry. b. The MDR-1 activity of the indicated Th subset assessed using multicolor flow cytometry with rhodamine 123 (Rh-123). Total CD4+ T cells isolated from the peripheral blood were labeled with Rh-123. After a 1-h efflux period at 37 °C in the presence of vehicle (dimethyl sulfoxide) or MDR-1 inhibitors (cyclosporine A), cells were stained with antibodies to CCR6, CXCR3, and CD45RO, and the Rh-123 efflux of each Th subset was analyzed using flow cytometry. Data shown are flow cytometry plots representing three independent experiments performed on cells isolated from different donors with RA. c. The proportion of cells with CD28 expression within the indicated CD4+ T cell subpopulations of patients with RA. Data are representative of at least three independent experiments.

We analyzed the subtype of peripheral blood T cells before and four weeks after ABA treatment (Fig 1a). Furthermore, in each cell group, Ki67 expression was determined by flow cytometry as a cell proliferation marker.

Th17.1 was the smallest subset of CD4+ cells (median, 1.17%; interquartile range, 0.71–1.93) (S1 Fig). Next, MDR-1 expression, one of the major features of Th17.1, was confirmed using Rh-123 (Fig 1b). The results showed that MDR-1 was highly expressed only in Th17.1 cells and not in Th1 and Th17 cells, as reported so far. The expression rate of CD28, an inhibitory target in ABA treatment, in the Th17.1 cells was as high as that in the others (>99%) (Fig 1c).

### Difference between early changes in the proliferation status of Th subsets

**Fig 2:**
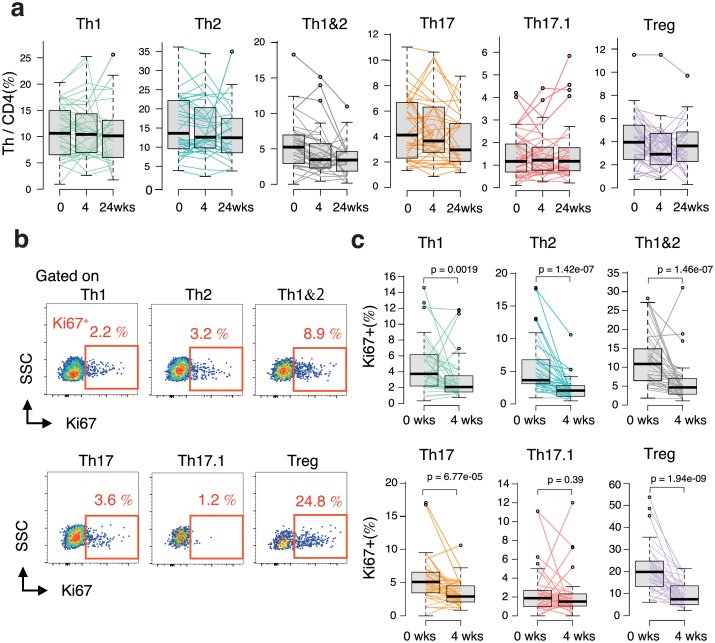
Early change in the cell proliferation state of each Th subset by ABA treatment. The peripheral blood mononuclear cells of patients with RA were obtained at baseline and 4 and 24 weeks of ABA treatment (0 and 4 weeks, n = 40; 24 weeks, n = 29). a. Sequential changes in the proportion of each T cell subset among CD4 T cells in the peripheral blood induced by ABA treatment. Data were analyzed using the Friedman rank sum test. b. Flow cytometry plot showing the frequency of Ki67 expression in the indicated CD4+ Th cells in patients with RA assessed using the intracellular staining of Ki67 antigen and analyzed using multicolor flow cytometry. c. Sequential changes in the proportion of Ki67 expression in each Th subset induced by ABA treatment (0 and 4 weeks, n = 40). Data were analyzed using the Wilcoxon signed rank test.

Changes in the proportions of Th subset among CD4+ T cells before and 4 weeks after ABA treatment were confirmed to evaluate the effect of ABA treatment on each Th subset. However, noticeable changes were not observed (Fig 2a). Therefore, we next analyzed Ki67 expression in the cells to confirm the early effects of ABA on each Th cell subset (Fig 2b). Before ABA treatment, the proportion of Ki67 positive cells among each Th subset was different; particularly, the expression rate in Th17.1 cells was remarkably lower than that in the other subsets (S2 Fig, Fig 2b and 2c). In contrast, the Ki67 expression rate in Tregs was relatively higher. Next, the Ki67 positivity rate for each Th subset was determined after ABA treatment for 4 weeks and compared with the baseline value. In only 4 weeks, the proportion of Ki67 positive cells was significantly reduced in all subsets other than the Th17.1 cells (Fig 2c). The change in Ki67 expression in Th17.1 cells was not statistically significant (p = 0.39).

### Therapeutic response to ABA and baseline Th17.1

**Fig 3:**
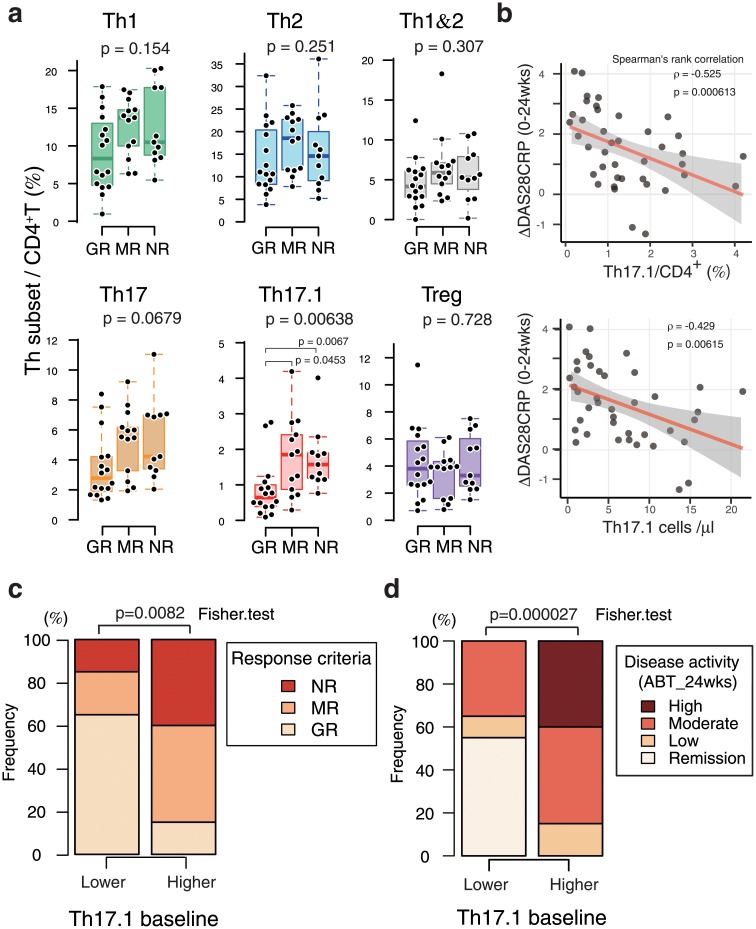
Clinical significance of Th17.1 levels in abatacept treatment response. ABA treatment was continued for 24 weeks. Subsequently, the correlation between the ABA response and proportion of Th subset at baseline was analyzed. The following indicators were used to evaluate response to ABA treatment: changes in the DAS28-CRP scores from baseline at 24 weeks after ABA treatment (ΔDAS28-CRP 24 weeks), disease activity evaluation after 24-week ABA treatment (remission and low, moderate, and high disease activities [LDA, MDA, and HDA, respectively]), and treatment response evaluation using the EULAR response criteria (good responder (GR), moderate responder (MR), and nonresponder [NR]). a. ABA treatment response after 24 weeks was evaluated as GR (n = 14), MR (n = 13), and NR (n = 13). The proportion of the indicated Th subsets among CD4+ lymphocytes at baseline in each group was plotted and displayed as box plot. b. Scatterplot shows the ratio or absolute number of Th17.1 cells at baseline and ΔDAS28-CRP 24 weeks. The regression line (red line) and its 95% CI (gray zone) are also shown in the plot. c. d. Patients were stratified into two groups (lower and higher) based on the median proportion of Th17.1 cells. The 100% stacked bar chart shows the EULAR response rate (c) and disease activity score (d) at 24 weeks after the initiation of ABA treatment in three groups. Data were analyzed using the Kruskal–Wallis and Mann–Whitney tests for between-group comparisons with Bonferroni correction (a), Spearman’s rank correlation coefficient (b), and Fisher’s exact test and Bonferroni correction for paired comparisons (c, d).

ABA treatment was continued for 24 weeks, and the progress of disease activity in each patient and responsiveness to treatment were evaluated. Subsequently, we analyzed the correlation between the ABA response and proportions of Th subset at baseline. A remarkable finding was that the proportion of baseline Th17.1 cells among CD4+ T cells in good responders was significantly lower than that in poor responders (p = 0.0064) (Fig 3a). In contrast, no significant difference was observed with respect to the other Th subsets.

The attenuation of disease activity (ΔDAS28-CRP) after 24 weeks of ABA treatment also showed a significant negative correlation with Th17.1 (both percentage and absolute number) (Fig 3b).

To assess the clinical relevance of the correlation between the proportion of Th17.1 cells and ABA response, we divided the patients into two groups (lower and higher) using the median Th17.1 proportion (/CD4+) as cutoff (Table 2). ABA treatment response in each group was analyzed. Treatment response was significantly different in the two groups (p = 0.0082) (Fig 3c). Treatment response in the Th17.1-lower group was significantly better than that in the higher group (S3a and S3b Fig). An analysis of the trend of disease activity at 24 weeks showed an association between a lower proportion of Th17.1 cells at baseline and higher percentage of remission. The remission rate in the Th17.1-lower group was 55%, whereas no remission was observed in the higher group (Fig 3d, S3b Fig).

**Table 2:** Clinical characteristics of the Th17.1-lower and Th17.1-higher patients at baseline.

Patient characteristics	Th17.1/CD4+ T cells		
	Lower n = 20	Higher n = 20	p
Age, year old	70.5 [60.8, 74.2]	71.0 [63.4, 74.7]	1.00
Sex: male/female, (%)	6/14 (30.0/70.0)	2/18 (10.0/90.0)	0.24
Disease duration (years)	3.4 [0.9, 6.4]	6.7 [2.5, 19.1]	0.07
DAS28-CRP	4.50 [3.77, 4.91]	4.43 [4.22, 5.02]	0.71
SDAI score	22.8 [18.5, 28.7]	24.3 [21.6, 29.2]	0.31
CRP, mg/dL	1.18 [0.57, 2.52]	0.82 [0.48, 2.16]	0.81
MMP-3 (ng/mL)	206.3 [104.6, 316.1]	152.2 [94.8, 267.8]	0.50
ACPA (negative/positive, [%])	9/11 (45.0/55.0)	7/13 (35.0/65.0)	0.75
Negative, (%)	9 (45.0)	7 (35.0)	0.67
Low positive, (%)	3 (15.0)	2 (10.0)	
High positive, (%)	8 (40.0)	11 (55.0)	
RF (negative/positive, [%])	7/13 (35.0/65.0)	7/13 (35.0/65.0)	1.00
Negative, (%)	7 (35.0)	7 (35.0)	1.00
Low positive, (%)	5 (25.0)	4 (20.0)	
High positive, (%)	8 (40.0)	9 (45.0)	
Concomitant methotrexate, n (%)	17 (85.0)	14 (70.0)	0.45
MTX, mg (mean (sd))	10.2 (2.5)	8.3 (3.1)	0.07
Concomitant glucocorticoid, n (%)	13 (65.0)	11 (55.0)	0.75
Prednisolone, mg (mean, [sd])	5.5 (4.3)	6.1 (3.0)	0.72
Concomitant tacrolimus, n (%)	0 (0.0)	3 (15.0)	0.23
Concomitant NSAIDs, n (%)	10 (50.0)	10 (50.0)	1.00
Biologic DMARDs naïve, n (%)	14 (70.0)	13 (65.0)	1.00
Pulmonary complications associated with RA, n (%)	7 (35.0)	11 (55.0)	0.34

Enrolled patients (n = 40) were stratified into two groups based on the median proportion of Th17.1 cells among CD4+ T cells: Th17.1 lower (n = 20) and Th17.1 higher (n = 20). The table shows the clinical features and differences of the Th17.1-lower and Th17.1-higher patient subgroups at baseline. Data are presented as median (IQR, interquartile range) or mean (SD), or frequency (%).

DAS28-CRP, disease activity score 28-joint count C-reactive protein; SDAI, Simplified Disease Activity Index; CRP, C-reactive protein; NSAIDs, nonsteroidal anti-inflammatory drugs; MMP-3, matrix metalloproteinase 3; ACPA, anti-citrullinated protein antibody; RF, rheumatoid factor; MTX, methotrexate; DMARDs, disease-modifying antirheumatic drugs; low positive, less than three times the normal upper limit among positive; high positive, more than three times the normal upper limit.

Between-group differences with respect to the median and percentage values were determined using the Mann–Whitney U and Fisher’s exact tests, respectively.

We assessed whether changes in the levels of the objective biomarkers of arthritis (serum CRP, MMP-3) after ABA treatment for 24 weeks were different between the Th17.1-lower and Th17.1-higher groups. A significant reduction in the serum CRP (S4a Fig) and MMP-3 (S4b Fig) levels was observed in the Th17.1-lower group; however, the reduction in these levels in the Th17.1-higher group was nonsignificant at 24 weeks. Furthermore, the serum CRP levels at 12 (p = 0.0496; S4a Fig) and 24 (p = 0.014; S4a Fig) weeks as well as the proportion of individuals with high titer of serum MMP-3 at 24 weeks were significantly higher in the Th17.1-higher group than in the Th17.1-lower group (p = 0.004; S4c Fig).

Subsequently, ROC curve analysis was performed to determine the optimal threshold level of the proportion of Th17.1 associated with good response or remission at 24 weeks (S5 Fig). A cutoff level of 1.09% (Th17.1 cells/CD4+ cells) was associated with 79.2% sensitivity and 81.2% specificity for GR and 75.9% sensitivity and 100% specificity for remission.

### Th17.1 and patient background factors

Differences in the clinical features between the Th17.1-lower and Th17.1-higher groups at baseline were evaluated; however, no significant differences were observed. Next, because CD4+ T cells play a significant role in RA pathogenesis, the correlation between various the patient characteristics, disease activity, proportion of Th subset, and ABA therapeutic response was assessed using Spearman’s rank correlation coefficient (S6 Fig). The baseline disease activity (DAS28-CRP baseline) showed a strong correlation with the serum CRP, ACPA, RF, and MMP-3 levels. In the Th subset, although baseline disease activity showed a negative correlation with the proportion of Treg, no significant correlation was observed with other Th subsets. The disease duration of RA showed a positive correlation between Th1&2, Th17, and Th17.1 cells. In the Th subset analysis, the proportion of Th17.1 showed a strong correlation with Th1 and Th17. The analysis of the correlation between patient characteristics and ABA response revealed a strong correlation of ΔDAS28-CRP (0–24 weeks) with baseline DAS28-CRP and age. However, none of the patient characteristics showed a significant correlation with the EULAR response criteria and disease activity after 24 weeks (S6 Fig, S1 Table). Therefore, in this study, we found no meaningful association between the background characteristics of patients and therapeutic response to ABA. However, the baseline proportions of Th17.1 and Th17 subsets showed a significant association with all three indices of ABA response (EULAR response criteria, ΔDAS28-CRP at 0–24 weeks, and RA disease activity score), and the baseline Th1 level was significantly associated only with disease activity after 24 weeks. Among these three Th subsets, the proportion of Th17.1 subset showed the most significant association with ABA response. The baseline Th17.1 levels in the GR group were more likely to be lower than those in the non-GR group. Regarding the proportion of Th17.1 at baseline in ACPA-high-positive patients, we found that the baseline Th17.1 levels were lower in the GR group than in the non-GR group (p = 0.0283) (S7 Fig). The same trends were observed in ACPA-negative and ACPA-low-positive patients, although the differences were not statistically significant.

Among the patient background factors and proportions of Th subsets, we selected and narrowed down the candidate variables to construct an optimal model for prognostic prediction using stepwise variable selection in multivariate analysis (S2 Table). In all multivariate analyses, only the proportion of Th17.1 was selected and showed the most significant association after adjustment for potential confounders. To evaluate the generalizability of this Th17.1-ABA model to independent cohorts, we performed leave-one-out cross validation (S3 Table). The concordance rate obtained using this cross validation was 78% (95% CI, 62–89), and the kappa coefficient was 0.54 (95% CI, 0.27–0.80). These results showed a moderate agreement between Th17.1 and response to ABA treatment. Moreover, the model using Th17.1 was not selected by overfitting.

**Fig 4:**
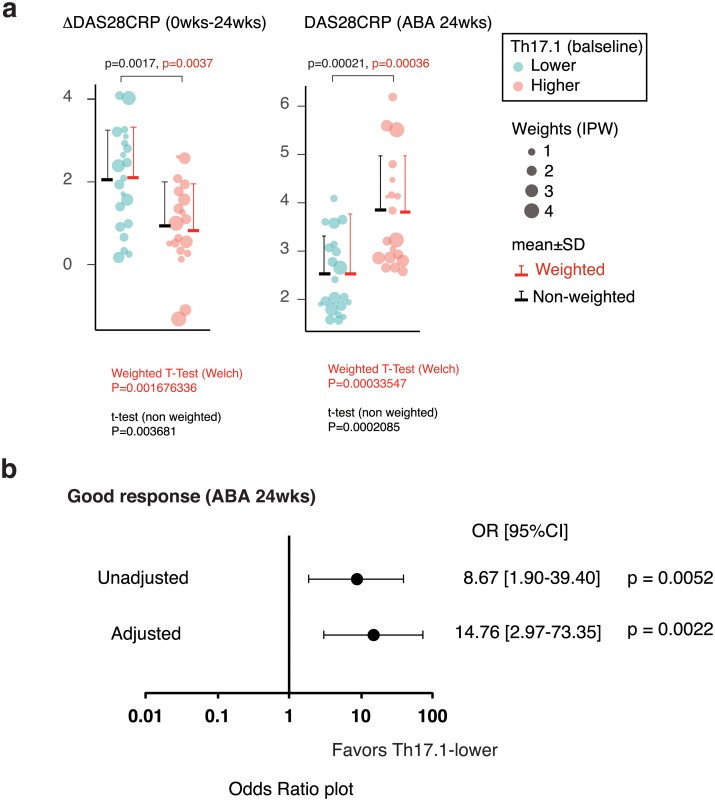
Prediction of therapeutic response to ABA based on the proportion of Th17.1 at baseline. a. The difference in ABA therapeutic response between the Th17.1-lower (binary by median) and Th17.1-higher groups after the adjustment of patient background factors using inverse probability weighting (IPW). The size of the balloon plot indicates the weighting using the IPW method in each case. The red lines indicate the weighted mean (horizontal line) and SD (vertical line) after IPW adjustment. Moreover, the black lines indicate the non-weighted mean (horizontal line) and SD (vertical line). Data were analyzed using the weighted (red) or non-weighted (black) *t*-test. b. The adjusted odds ratio for the achievement of GR with baseline Th17.1-lower relative to Th17.1-higher. Logistic regression analysis was conducted using the IPW method to calculate the odds ratio adjusted for patient characteristics. Forest plot shows the unadjusted and adjusted odds ratios and 95% CI and P value. ABA, abatacept; IPW, inverse probability weighting; SD, standard deviation; OR, odds ratio; CI, confidence interval.

Given the limited number of cases for adjusting confounding factors by multivariate analysis, the IPW method was used to reduce the number of confounders and analyze the adjusted effect of the baseline proportion of Th17.1 on ABA therapeutic response. The C-statistic, the discriminative power of PS for the Th17.1-lower group was 0.735 (95% CI, 0.576–0.894). With IPW, all patient background factors that are shown in S4 Table were more evenly adjusted between Th17.1-lower and Th17.1-higher, including RA disease duration, which was not significantly different but tended to correlate. Of note, even after adjustment for different covariate distributions for both groups, a significant difference was noted between the Th17.1-lower and Th17.1-higher groups with respect to ΔDAS28-CRP (0–24 weeks) and disease activity after 24 weeks (DAS28-CRP 24 weeks) (Fig 4a). The effect of Th17.1-lower on ABA good response as compared with that of Th17.1-higher was evaluated by estimated OR with 95% CIs, after adjustment by IPW. In this study, GRs and patients with low disease activity after 24 weeks were equivalent. As a result, in the Th17.1-lower group, the OR for achieving good response was 14.6 (95% CI, 2.9–72.3; p = 0.0021) (Fig 4b, S5 Table). The proportion of Th17.1 cells among CD4+ T cells at baseline was a good predictor of ABA treatment response.

## Discussion

We found that baseline Th17.1 levels may be a prognostic predictor of ABA treatment in patients with RA. Moreover, although Th17 was correlated with therapeutic response to ABA, Th17.1 showed a stronger correlation. Among Th cells that induce antigen-specific responses, the identification of cells that are associated with ABA therapeutic response is the most novel finding of this study.

In this study, first, we analyzed Th17.1 in detail via flow cytometry. Next, intracellular Ki67 was stained to clearly show cell proliferation state. A key novelty aspect of this study was the analysis of cell surface CD161, intracellular forkhead box P3 (Foxp3), and Ki67 via flow cytometric analysis. Although CD161 is not a marker in the international standard human immunophenotyping method [28], it is a significant surface marker of pathogenic Th17 and Th17.1 subsets. In addition, CCR6 and CCR4 are expressed in Treg; therefore, intracellular Foxp3 was stained to precisely exclude Tregs. Ki67 expression analysis helped identify the early change in the cell proliferation status of each Th subset induced by ABA treatment.

Finally, we used IPW for adjusting patients’ background characteristics. Th17.1 was significantly correlated with these background characteristics, such as disease duration. Even after adjusting for the background characteristics using the IPW method, the baseline proportion of Th17.1 subset significantly predicted the ABA treatment response. To identify cellular immunological biomarkers that predict therapeutic response via flow cytometric analysis, the adjustment of patients’ background characteristics is essential because target cells themselves may be associated with these characteristics other than therapeutic effect. However, because of the limited number of samples owing to the handling of living cells and the associated cost burden, it is difficult to adjust for confounding factors via multivariate analysis. In such settings, using the IPW method to adjust for patients’ background characteristics is a well-accepted practice.

How does Th17, particularly the most pathogenic Th17.1, correlate with ABA treatment response? As a possibility, we consider the following.

The first possible reason is that ABA treatment response resistance is attributable to the presence of high levels of Th17.1 drivers such as inflammatory cytokines. Inflammatory cytokines such as IL-1β, IL-23, and IL-6, which are produced by dendritic cells, reportedly induce pathological Th17 and Th17.1 differentiation [29–31]. Moreover, saturated fatty acid content and salt intake reportedly accelerate Th17-mediated pathologies [14]. In patients with these Th17.1 drivers, the pathogenic T cells may not be sufficiently suppressed by ABA, although we did not investigate the levels of these Th17 drivers and the associated factors, which is a limitation of this study. Another possibility for the correlation between Th17.1 and ABA responsiveness is that Th17.1 cells themselves are a main factor in ABA treatment response resistance. Both Th17 and Th17.1 cells are present in the synovial fluid of patients with RA; however, the latter are particularly more abundant in the synovial fluid than in the peripheral blood [32]. Th17.1 cells are pathological inflammatory cells [12] and produce multiple pro-inflammatory cytokines, such as IL-17A, granulocyte-macrophage colony-stimulating factor (GM-CSF), interferon-γ, and TNF-α, which are associated with rheumatoid inflammatory conditions. Treatment with a neutralizing antibody against GM-CSF, which is produced in high amounts by Th17.1, is effective in patients with RA [33]. Further, Ki67 positivity rate in Th17.1 hardly decreased in the early stage of ABA treatment, indicating that Th17.1 is less susceptible to ABA regarding cell proliferation. By combining these previous reports with our results, it can be inferred that Th17.1 is an important inflammatory cell group that is abundant in the inflammatory joints of patients with RA and poorly responsive to ABA. This supports the possibility that Th17.1 is resistant to ABA treatment and that Th17.1 cells play a significant role in RA activity even during ABA treatment in NRs. Targeting Th17.1 by either suppressing the function or depleting the cells may be required to suppress the disease activity in patients with high numbers of Th17.1 cells.

Why are pathological Th17 and Th17.1, but not Th1, inversely correlated with the therapeutic response to ABA? The possible reason is the difference in the response of Th17 and Th17.1 to co-stimulatory and co-inhibitory signals from those of other Th subsets. Unlike that of other Th subsets, pathological Th17 differentiation is inhibited by CD28 co-stimulation [31,34], suggesting that CD28 co-stimulatory signal inhibition by ABA treatment does not suppress the inflammatory cytokine-induced Th17 differentiation in patients with RA. Furthermore, the balance between the co-stimulatory signals from CD28 and co-inhibitory signals from CTLA-4 in each Th cell subset after ABA treatment may be significant. Although co-stimulation by CD28 on T cells is essential for modulating the T-cell immune response in autoimmune diseases, inhibitory pathways by CTLA-4 on T cells are also critical [35]. It is known that the interaction between CTLA-4 on Th17 cells and B7 inhibits Th17 differentiation and suppresses Th17-mediated autoimmune myocarditis in mice [36]. It can be inferred that the binding of ABA to B7 molecules inhibits co-inhibitory pathway via CTLA-4 expressed on T cells in patients with RA. Interestingly, a previous report [37] has shown that in human renal transplant patients, the memory phenotype of Th17 expresses CTLA-4 at a higher level than that expressed by other Th subsets. This increases the sensitivity of belatacept, which has a mechanism of action similar to that of ABA, to Th17 cell co-inhibitory signals. Moreover, renal transplant patients with increased Th17 cell numbers showed resistance to belatacept. These findings indicate that pathogenic Th17 and Th17.1 may exacerbate immune diseases through the ABA treatment-mediated inhibition of the co-stimulatory signals from CD28 and co-inhibitory signals from CTLA-4 in patients with RA, although we did not investigate CTLA-4 levels on Th17.1, which is a limitation of this study. Further investigations are needed to determine the biological mechanism of ABA treatment resistance by Th17.1.

The proportion of Treg at baseline in the 40 patients in this study varied widely (median, 3.9%; interquartile range, 2.5%–5.4%; minimum, 0.74%; and maximum, 11.5%). However, in contrast to Th17.1 cells, these Treg differences did not predict the response to ABA treatment. The immunological mechanisms of RA pathology, such as the disruption of autoimmune tolerance, onset and persistence of inflammation, and joint destruction, are extremely complex. Even in the contribution of T cells to the persistence of inflammation, the significance of the involvement of dysfunction in the suppression (regulatory) side, such as Treg, and the existence of inflammatory T cells that can resist Treg suppression is also conceivable. Treg expresses cytotoxic T-lymphocyte antigen-4 (CTLA-4) [38], which is an immunosuppressive functional molecule common to both Treg and ABA. It has been inferred that immune system inactivation by CTLA-4-Ig strongly supplements the difference in the amount of Treg and that the influence of endogenous Treg in arthritis is reduced. In contrast, when inflammatory cells that are resistant to suppression by these CTLA-4 molecules are present at baseline, the levels of these inflammatory cells are likely to affect disease activity after ABA treatment. CTLA-4 molecule is a strong negative regulator of T cell immune response [39,40] and plays a central role in Treg’s regulatory function [38]. More interestingly, it has been recently reported that ex-Th17, having the same phenotype as Th17.1, is not restricted by Treg suppression [41]. On the basis of these facts and the results of this study, we determined that Th17.1 cells play a role in disrupting immune tolerance by CTLA-4 and Treg. Further biological studies must be conducted to assess whether Th17.1 is resistant to not only Tregs but also CTLA-4-Ig suppression.

Previous studies have reported only few factors that adequately predict ABA treatment response in patients with RA. These include CRP [42], presence or absence of MTX combination, history of use of other biologics [43], positivity for ACPA and RF [44], and age [45]. In this study, no significant correlation was observed between these known baseline patient characteristics and the proportion of peripheral blood Th17.1 cells. None of the known patient characteristics were found to predict ABA treatment response. Furthermore, the following lymphocyte-related indices, which are independent of Th17.1, were reported as prognostic factors: proportion of terminally differentiated effector memory cells among CD8 T cells [46] and that of CD28-negative T cells [47,48]. However, these lymphocyte subsets were not analyzed in this study. Therefore, the correlation between these known lymphocytes and Th17.1 was not clarified, which is a study limitation.

This was an exploratory study; we did not evaluate the generalization performance of Th17.1 as a biomarker in another prospective cohort. Thus, this is a limitation of this research. However, using leave-one-out cross validation, we showed that the model in this study is not likely to be overfitting.

Although the choice of treatment for patients with RA who are refractory to MTX treatment is of significant importance for clinicians, it may be challenging to decide the treatment on the basis of the results of this study alone. Baseline Th17.1 was positively correlated with the duration of the disease, implying a trend toward better ABA treatment response in patients with early RA. Further clinical studies are needed to elucidate the correlation of disease duration and proportion of Th17.1 with ABA treatment response. Various treatment options are available in the clinical practice of RA; however, it is significant to promote personalized medicine. Because Th17.1 produces pro-inflammatory cytokines, further research is needed to determine whether it is a predictor of therapeutic response to not only ABA but also cytokine therapeutic agents, such as TNF-α inhibitors, IL-6 receptor antibody, GM-CSF inhibitor, and Janus kinase inhibitors. Although various biomarkers are available for predicting therapeutic response, they are insufficient for the vast majority of diseases. Therefore, further studies on precision medicine must be conducted.

## Conclusions

The present study demonstrated that the proportion of circulating Th17.1 cells showed differences in immunological quality that determine the therapeutic response to ABA in patients with RA. A vast array of antirheumatic drugs are currently available for the treatment of RA. The identification of the most appropriate drug for individual patients is key imperative to achieve early improvement. The identification of Th17.1 as a good candidate biomarker of the therapeutic response to ABA may represent a significant step in the pursuit of precision medicine.

## Supporting information

S1 Fig The proportion of cells with Ki67 expression among circulating CD4+ Th subsets in patients with RA. PBMCs from RA (n = 26, before abatacept treatment) and healthy controls (n = 15) were stained for CD4, CXCR3, CCR4, CD161, CCR6, CD25, Foxp3, and Ki67 mAbs and analyzed using flow cytometry. The percentage of Ki67+ cells in the indicated Th subsets are shown by box plot. Data were analyzed using the Kruskal–Wallis test, followed by the Mann–Whitney U test using Bonferroni correction.
(EPS)

S2 Fig Changes in the cell proliferation state of Th17 and Th17.1 subsets by ABA treatment. The graph shows changes in the proportion of cells with Ki67 expression among each Th subset induced by ABA treatment at various time points (0, 4, and 24 weeks; n = 29). Data were analyzed using the Friedman rank sum test. The Wilcoxon signed rank test with Bonferroni correction was used for *post hoc* paired comparisons.
(EPS)

S3 Fig Th17.1 level and successive changes in the disease activity score. **a**. The line graph shows the transition of the disease activity (DAS28-CRP) of RA in the Th17.1-lower and Th17.1-higher groups before and after ABA treatment (at 4, 12, and 24 weeks). P values (vs. Th17.1-higher) were determined with the Mann–Whitney U test using the Bonferroni correction. **b**. The 100% stacked bar chart shows successive changes in DAS28-CRP in the Th17.1-lower and Th17.1-higher groups before and after ABA treatment (at 4, 12, and 24 weeks). ABA, abatacept; DAS28-CRP, disease activity score 28-joint count C-reactive protein; REM, remission; LDA, low disease activity; MDA, moderate disease activity; HDA, high disease activity.
(EPS)

S4 Fig Th17.1 level and successive changes in the CRP and MMP-3 levels. **a. b**. The line graphs show the transition of serum C-reactive protein (CRP) and metalloproteinase-3 (MMP-3) of rheumatoid arthritis in the Th17.1-lower and Th17.1-higher groups before and after ABA treatment (at 4, 12, and 24 weeks). Data were analyzed using the Mann–Whitney tests for the comparisons of the Th17.1-lower and Th17.1-higher groups. The Wilcoxon signed rank test with multiple comparisons using the Bonferroni correction were conducted to analyze the sequential changes in the serum CRP and MMP-3 levels. **c**. The 100% stacked bar chart shows MMP-3 titer (normal, moderate, and high) in the Th17.1-lower and Th17.1-higher groups after ABA treatment at 24 weeks. P values (Th17.1-lower vs. Th17.1-higher) were determined using Fisher’s exact test. ABA, abatacept; CRP, C-reactive protein; MMP-3, metalloproteinase-3; normal, within normal limit; moderate titer, less than three times the normal upper limit; high titer, more than three times the normal upper limit.
(EPS)

S5 Fig Estimation of Th17.1 cutoff value at baseline to predict ABA therapeutic response using the ROC curve. **a**. ROC curve showing a Th17.1 (% in CD4+) cutoff level of 1.1% discriminated between GR and non-GR (MR or NR) at 24 weeks, with 79.2% sensitivity and 81.2% specificity. **b**. ROC curve showing a Th17.1 cutoff level of 1.1% discriminated between REM and non-REM at 24 weeks, with 75.9% sensitivity and 100% specificity. ROC, receiver operating characteristic; AUC, area under the curve; GR, good response; MR, moderate response; NR, no response; REM, remission.
(EPS)

S6 Fig The correlation coefficient matrix plot shows the correlation (Spearman’s correlation coefficient, ρ) of patient background factors, indicated T cell subset at baseline, and ABA therapeutic response indicators with significance levels (P value). (EPS)

S7 Fig Low proportion of Th17.1 cells in good responders regardless of the ACPA levels. Enrolled patients (n = 40) were stratified into three groups based on the ACPA levels (ACPA high positive, n = 19; ACPA low positive, n = 5; and ACPA negative, n = 16). Each group was further divided into two groups of GR and non-GR in response to ABA treatment, and the proportion of Th17. 1 among CD4+ T cells is shown. P values (GR vs. non-GR) were determined using the Mann–Whitney U test. GR, good responder (EULAR response criteria); non-GR, non-good responder (moderate responder or nonresponder); ACPA, anti-citrullinated protein antibody; low positive, less than three times the normal upper limit among positive; high positive, more than three times the normal upper limit.
(EPS)

S1 Table Differences in baseline clinical characteristics between EULAR-GR and non-GR patients. (DOCX)

S2 Table Exploratory analysis for optimal Th subset as the predictor of ABA treatment response using multivariate analysis. (DOCX)

S3 Table Leave-one-out cross validation of the Th17.1-ABA model. (DOCX)

S4 Table Adjusted characteristics of the Th17.1-lower and Th17.1-higher patients using IPW. (DOCX)

S5 Table Logistic regression analysis using the IPW method to calculate the odds ratio adjusted for patient characteristics. (DOCX)
