# Supplementary material for: Circulating Th17.1 cells as candidate for the prediction of therapeutic response to abatacept in patients with rheumatoid arthritis: An exploratory research
Source: PLoS One. 2019 Nov 20;14(11):e0215192. doi: 10.1371/journal.pone.0215192 (PMC6867595; doi:10.1371/journal.pone.0215192)
Supplement: S1 Table — (DOCX) [file pone.0215192.s008.docx]

- **S1 Table. Differences in baseline clinical characteristics between EULAR-GR and non-GR patients.**
-
- Data are presented as median (IQR, interquartile range), mean (SD), or frequency (%).
- EULAR, European League Against Rheumatism; GR, good response; MR, moderate response; NR, no response; DAS28-CRP, disease activity score 28-joint count C reactive protein; SDAI, simplified disease activity index; CRP, C-reactive protein; NSAIDs, non-steroidal anti-inflammatory drugs; MMP-3, matrix metallo-proteinase 3; ACPA, anti-citrullinated protein antibody; RF, rheumatoid factor; MTX, methotrexate; DMARDs, disease modified anti-rheumatic-drug; Low positive, less than 3 times normal upper limit among positive; High positive, more than 3 times the normal upper limit.
- Between-group differences with respect to median values were determined using Mann–Whitney U test, and those with respect to percentage values were assessed using Fisher’s exact test.
