# Supplementary material for: Circulating Th17.1 cells as candidate for the prediction of therapeutic response to abatacept in patients with rheumatoid arthritis: An exploratory research
Source: PLoS One. 2019 Nov 20;14(11):e0215192. doi: 10.1371/journal.pone.0215192 (PMC6867595; doi:10.1371/journal.pone.0215192)
Supplement: S2 Table — (DOCX) [file pone.0215192.s009.docx]

- **S2 Table**. **Exploratory analysis for optimal Th subset as the predictor of ABA treatment response using multivariate analysis.**

- The following analyses were performed to determine the candidate Th subset that predicted ABA response: Multivariate analysis was performed with the Th subsets or Th subsets and patient background factors as the predictor variable and ABA response [ΔDAS28-CRP (0–24 weeks); multiple regression (A, C), EULAR Response-GR; logistic analysis (B, D)] as the outcome variable. The candidate predictor variable was selected using the stepwise variable selection method based on AIC, BIC, and p-value. First, only Th subsets (Th1, Th2, Th1&2, Th17, Th17.1, and Treg) were used as predictor variable (A, B). In all analyses (AIC, BIC, and p-value method), only Th17.1 was found to predict ABA response. Next, Th subsets and patient background factors were used as predictor variable (explanatory variables) (C, D). In both analyses, only Th17.1 showed a significant association with ABA response. Among other background factors, the history of biological DMARDs and disease activity at baseline remained as candidates.
- ABA, abatacept; AIC, Akaike's Information Criterion; BIC, Bayesian Information Criterion
