# Supplementary material for: Circulating Th17.1 cells as candidate for the prediction of therapeutic response to abatacept in patients with rheumatoid arthritis: An exploratory research
Source: PLoS One. 2019 Nov 20;14(11):e0215192. doi: 10.1371/journal.pone.0215192 (PMC6867595; doi:10.1371/journal.pone.0215192)
Supplement: S3 Table — (DOCX) [file pone.0215192.s010.docx]

- **S3 Table. Leave-one-out cross validation of Th17.1-ABA model.**

A

B

Leave-one-out cross validation were performed to validate Th17.1-ABA model (A). For each combination of test and training cases, we show the cut-off value of Th17.1 determined by the training cases and the actual and predicted responses (GR if below the cut-off) of the test case. A confusion matrix between the actual and predicted responses are also presented (B).

- ABA, abatacept; ROC, receiver operating characteristic; AUC, area under the curve; GR, good response; non-GR, non-good response (moderate response or no response)
