# Supplementary material for: Circulating Th17.1 cells as candidate for the prediction of therapeutic response to abatacept in patients with rheumatoid arthritis: An exploratory research
Source: PLoS One. 2019 Nov 20;14(11):e0215192. doi: 10.1371/journal.pone.0215192 (PMC6867595; doi:10.1371/journal.pone.0215192)
Supplement: S4 Table — (DOCX) [file pone.0215192.s011.docx]

- **S4 Table. Adjusted patient characteristics of Th17.1-lower and Th17.1-higher patients by IPW**

- Using the patient background factors, we calculated the propensity score (PS) to become the Th17.1-lower group. For the estimation of PS, we used a logistic regression model in which the Th17.1 status (lower or higher) was regressed on the following baseline patient background factors: age, sex, DAS28-CRP (baseline), RF, ACPA, RA disease duration (years), biological DMARDs history, and prescription of methotrexate, and glucocorticoids. Next, all patient background variables between Th17.1-lower and Th17.1-higher groups were adjusted using the inverse probability weighting (IPW) method. Adjusted results are weighted mean [± standard deviation (SD)] and weighted median [interquartile range (IQR)]. Non-adjusted results are mean ± SD and median (IQR). Data were analyzed by *t*-test, Mann–Whitney test, or chi-squared test (non-adjusted results) or weighted *t*-test, weighted Mann–Whitney test, or weighted chi-squared test (adjusted results).
- RA, rheumatoid arthritis; RF, rheumatoid factor; ACPA, anti-citrullinated protein/peptide antibody; DAS28-CRP, disease activity score 28-joint count C-reactive protein; PS, propensity score; IPW, inverse probability weighting; SD, standard deviation; IQR, interquartile range; DMARDs, disease modified anti-rheumatic-drug**.**
