# Supplementary material for: Circulating Th17.1 cells as candidate for the prediction of therapeutic response to abatacept in patients with rheumatoid arthritis: An exploratory research
Source: PLoS One. 2019 Nov 20;14(11):e0215192. doi: 10.1371/journal.pone.0215192 (PMC6867595; doi:10.1371/journal.pone.0215192)
Supplement: S5 Table — (DOCX) [file pone.0215192.s012.docx]

- **S5 Table**. **Logistic regression analysis using the IPW method to calculate odds ratio adjusted for patient characteristics**.

|  |  |  |  |  |  |  |  |
| --- | --- | --- | --- | --- | --- | --- | --- |
|  |  | β | Std. Error | Odds ratio | 95% CI | Waldχ2 | p |
|  |  |  |  |  |  |  |  |
| Unadjusted | (Intercept) | −1.67 | 0.63 | - | - | - | - |
|  | Th17.1-lower | 2.16 | 0.77 | 8.67 | 1.90–39.40 | 7.8 | 0.0052 |
|  |  |  |  |  |  |  |  |
| IPW-adjusted | (Intercept) | −1.8 | 0.65 | - | - | - | - |
|  | Th17.1-lower | 2.69 | 0.82 | 14.76 | 2.97–73.35 | 10.83 | 0.0022 |
|  |  |  |  |  |  |  |  |

- The table shows unadjusted and IPW-adjusted odds ratios for achieving GR at week 24 with baseline Th17.1-lower relative to Th17.1-higher.

ABA, abatacept; GR, good response; IPW, inverse probability weighting
